# Reaching and maintaining higher dietary diversity is associated with decreased risk of all-cause mortality: A longitudinal study from the China Health and Nutrition Survey

**DOI:** 10.3389/fnut.2022.947290

**Published:** 2022-09-26

**Authors:** Xiaochen Qu, Xiaona Na, Jiaqi Yang, Haoran Yu, Aiwen Chen, Ai Zhao

**Affiliations:** ^1^Vanke School of Public Health, Tsinghua University, Beijing, China; ^2^Department of Nutrition and Food Studies, New York University, New York, NY, United States; ^3^Department of Psychology, Carnegie Mellon University, Pittsburgh, PA, United States

**Keywords:** dietary diversity, mortality, trajectory modeling, longitudinal study, China Health and Nutrition Survey

## Abstract

It is generally believed that higher dietary diversity is associated with better health status. The dietary diversity of individuals may change with age; however, evidence on the trajectory of change in the long-term and whether it is related to all-cause mortality is still scant. In this study, we used data from the China Health and Nutrition Survey (CHNS) collected in five follow-ups between 2004 and 2015 to explore the association between changes in dietary diversity scores (DDS) and all-cause mortality, as well as the dynamic change in DDS with age. In total, 6,737 subjects (aged between 30 and 60 at enrollment) were included in the analysis. Latent Class Trajectory Modeling (LCTM) was used to explore the different trajectories of DDS changes among participants. Four classes were identified: class 1 with the lowest average DDS (3.0) that showed a gradual decline during the follow-ups; class 2 with relatively low DDS (4.0) that experienced slight growth; class 3 with medium DDS (5.2) that also demonstrated similar growth rate to class 2; and class 4 with the highest DDS (6.7) maintained at a high level. Cox proportional hazards regression models were applied to investigate the association between the DDS trajectories and the risk of death. Only class 4, which was characterized by the highest and stable DDS, had significant reduced risk of all-cause mortality of 71.0% (hazard ratio *[HR]*: 0.29; 95% confidence interval *[CI]*: 0.10–0.83), 68% (*HR*: 0.32; 95% *CI*: 0.11–0.89), and 66.0% (*HR*: 0.34; 95% *CI*: 0.12–0.94), compared to classes 1, 2, and 3, respectively, while the first three classes showed no significant inter-class differences. When considering the average DDS during the study period, each point of increase in DDS corresponded to a 22% reduced risk of mortality (*HR*: 0.78; 95% *CI*: 0.69–0.89). In summary, reaching and maintaining a higher DDS was associated with a decreased risk of all-cause mortality. Therefore, promoting diversified eating and increasing the accessibility of varieties of foods should be paid more attention from policymakers and be more emphasized in dietary guidelines.

## Introduction

Consuming a variety of foods is one of the principles of dietary guidelines in many countries (1–3). The variation in food consumption can be measured by the dietary diversity score (DDS), reflecting nutrient adequacy and dietary quality of individuals (4). Previous studies showed that a higher DDS was related to reduced risks of obesity (5) and many age-related diseases, such as diabetes (6), cognitive, and memory status decline (7–9). It was suggested that higher dietary diversity was also inversely associated with all-cause mortality among elderly people (10) and people who enjoyed greater diversity of diet maintained a longer healthy life expectancy (11). However, another study showed mixed results, indicating that a higher DDS might only decrease mortality among females but not males (12). Therefore, whether DDS is associated with all-cause mortality, and how strong the association is in different populations remain unclear.

Furthermore, food intake and dietary diversity may change over the lifetime due to multiple reasons. With age, the loss of appetite decreased total energy intake, and a worse financial situation may lead to a higher risk of malnutrition (13, 14). Lower chewing ability and eating alone may also decrease the diversity of diet (15, 16). A study showed that dietary diversity significantly declined in women older than 63 years old, and a trend of decrease was also observed in men at the age of 65 years and higher, although not statistically significant (17). However, studies investigating the dynamic change of dietary diversity over time are still scant.

China, the country with one of the largest populations in the world, has experienced a rapid change to an aging society, accompanied by dietary patterns and transitions of its citizens due to the growth of the economy during the past decades (18, 19). Coming along with abundant food options, the prevalence of diet-related non-communicable diseases, such as hypertension, stroke, type-2 diabetes, coronary heart disease, as well as obesity are rising (18). Meanwhile, micronutrient deficiencies also exist, more than half of Chinese adults' micronutrient intake did not meet the Chinese estimated average requirement, and more than 80% of Chinese adults had two or more kinds of micronutrient deficiencies (20). Because of the triple burden of undernutrition, overnutrition, and micronutrient deficiency faced by Chinese adults, more effective dietary guidance that is easy to be followed by the public is urgently needed (21, 22). Increasing dietary diversity and maintaining a high-dietary diversity can be a feasible and effective way to increase nutrient adequacy and dietary quality.

To close the gaps between DDS changes and their associations with mortality, as well as provide valuable scientific evidence to improve the current dietary guidelines, the present study aims to investigate the long-term dynamic change in dietary diversity and its association with all-cause mortality using data from the China Health and Nutrition Survey (CHNS).

## Materials and methods

### Study design and population

**Figure 1:**
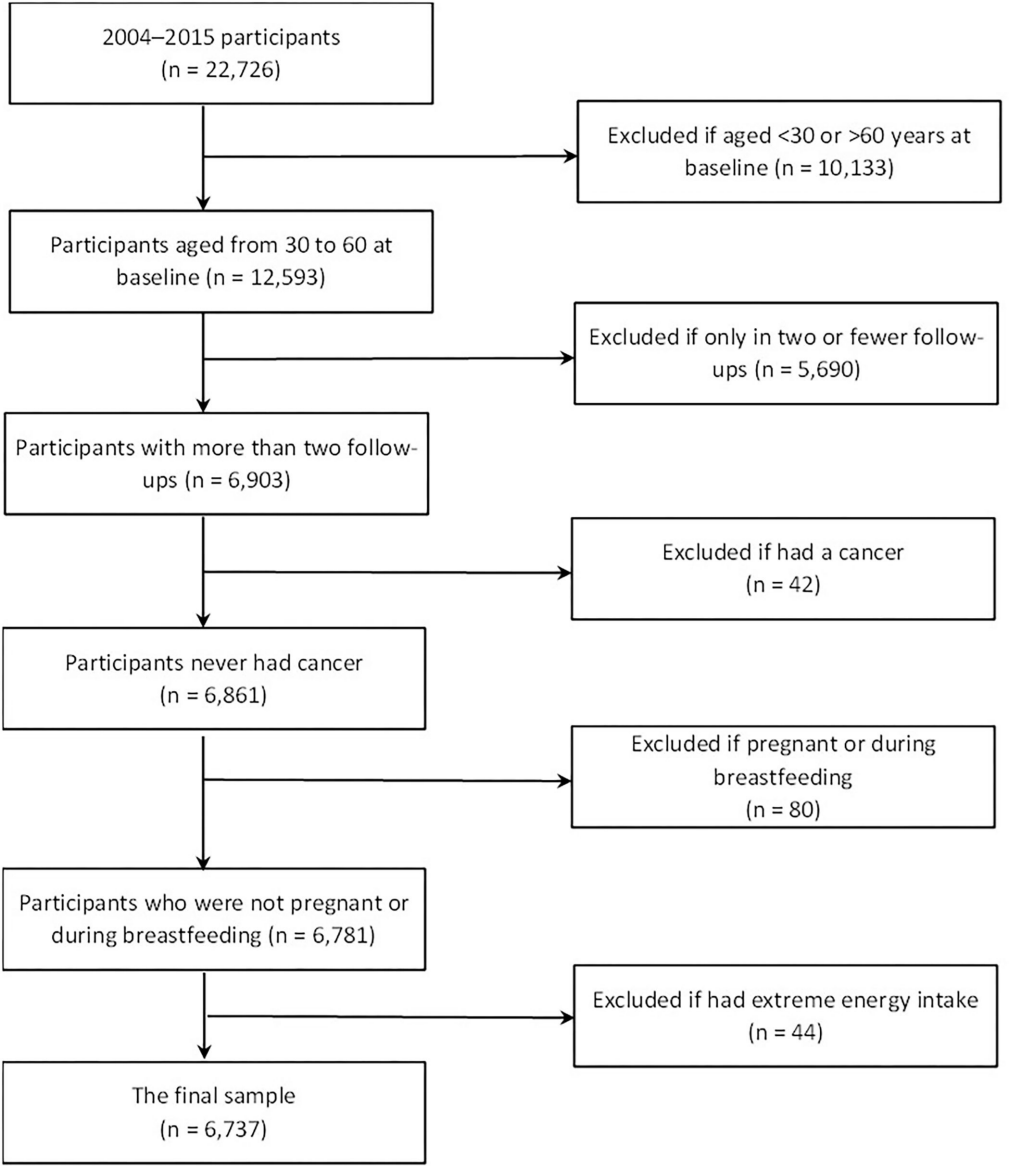
Flowchart of sample selection.

The CHNS is a nationwide prospective cohort study. The initial recruitment of participants was conducted in 1989, and follow-ups were conducted within a 2–3-year interval. Participants were recruited from nine provinces and three autonomous cities. The detailed description can be found elsewhere (23). In this study, we used the data from the CHNS collected in 2004, 2006, 2009, 2011, and 2015. Since the dietary data for 2015 are not yet available, only mortality data were included from that interval. To cover the dietary diversity changes in the aging process, and to avoid significant dietary restriction by diseases, we excluded the participants who were younger than 30 years old or older than 60 years old at baseline (age at baseline was identified as the actual age in the year of entering the cohort during 2004–2015 because the year of participants entering the cohort varied), pregnant or during breastfeeding, and those who only participated in two or fewer follow-ups, had cancers and extreme energy intake (< 500 kcal/day or >8,000 kcal/day) throughout the follow-ups period. A total of 6,737 participants were included in the analysis (Figure 1).

The CHNS was approved by institutional review boards at the University of North Carolina (Chapel Hill, NC, the US) and the National Institute of Nutrition and Food Safety (Chinese Center for Disease Control and Prevention). Informed consent was given to all participants before participation. The current study was further approved by the Institution Review Board of Tsinghua University (project identification 20210072).

### Dietary assessment and dietary diversity score

Dietary data at the individual level were collected using 24-h dietary recalls for consecutive 3 days. Participants were required to report all the food consumed at home and away from home over the past 24 h (24). The quality of dietary data was controlled in several ways. First, besides 24-h dietary recalls, changes in the inventory of certain food, such as rice, flour, edible oil, and condiments, were weighed and measured at the household level, which served as a validation of 24-h dietary recall. The significant discrepancies were resolved according to revisiting and investigating their food consumption. Second, all the interviewers were trained nutritionists who were professionally engaged in nutrition work and had participated in other national surveys. In addition, three days of specific training in the collection of dietary data have been provided for this survey (29). In total, 34 kinds of foods and 21 kinds of nutrients were recorded (25). Energy intakes of subjects were calculated by multiplying the consumption of each food and their macronutrients' content acquired from the Chinese Food Composition Tables (26–28). The detailed method of dietary assessment was described elsewhere (29).

DDS was developed based on Chinese dietary guidelines 2022 (CDG-2022) and Chinese Food Composition Tables (26–28, 30). Ten food groups are included in the CDG-2022: cereals and tubers, vegetables, fruits, meat, soybeans and nuts, eggs, aquatic products, milk, and dairy products, as well as salt and oil, in which salt and oil were not included in the DDS assessment (31). Particularly, mixed beans (dried legumes other than soybeans, such as azuki bean and mung bean) and tubers (such as potato and sweet potato) belong to the category of cereals and tubers. Fresh beans and tuber vegetables belong to the category of vegetables. Soybeans, nuts, and seeds belong to the category of soybeans and nuts (32). For each participant, the daily average DDS was calculated during each follow-up.

The trajectory of dietary diversity was analyzed by Latent class trajectory modeling (LCTM), which is a relatively new method in epidemiology studies. Populations with heterogeneous characteristics could be divided into several simplified homogeneous classes by this method. For the modeling process, we referred to the systematic framework introduced by Lennon et al. and adapted it for our study (33). The modeling contained six steps: (1) building a scoping model with five classes; (2) refining the number of classes; (3) refining model structure to a flexible random-effect specification; (4) model adequacy assessment; (5) graphical presentations; and (6) checking clinical characterization and plausibility, that is, the plausibility of DDS scores of each class and the changes over years.

The “lcmm” package (version 1.9.3) in R Statistical Software was used for modeling. The optimal number of classes was chosen based on the lowest Bayes information criterion (BIC) and the criterion that the number of participants in each class should not be less than 5% of the total participants (34). The trajectory model was selected by comparing the BIC and number of participants of models with (1) only fixed effects, (2) fixed effects and random intercepts, and (3) fixed effects and random slopes. The trajectory shapes (whether including cubic and quadratic terms) of the classes were determined according to the significant coefficients of model running results, and linear models were constructed if the polynomial coefficients were not significant (35). For the adequacy assessment, the model with the lowest BIC and the posterior probability of assignments (APPA) of each class ≥ 0.7 was selected as the final model.

### Outcome assessment

The primary outcome of the present study is all-cause mortality. For each participant in the CHNS, the household register system would continuously update their status, either alive or deceased, and the year and month of death. The year of follow-up was calculated from enrollment to the date of passed away or loss of follow-up of the participant during 2004–2015.

## Statistical analysis

Normality was examined by a combination of histogram and the Kolmogorov-Smirnov test because of the large sample size, and the data with an asymmetrically distributed histogram or *P* < 0.05 in the Kolmogorov–Smirnov test were defined as non-normality. Characteristics for all eligible participants are described as mean and standard deviation (SD; with a normal distribution) or median and interquartile range (*P*_25_*, P*_75_; without normal distribution) for continuous variables, and frequencies and percentages for categorical variables. Total person-years during the follow-ups were calculated by the sum of all the participants' total person-years participating in the survey during 2004–2015. The differences in sociodemographic characteristics, history of diseases, and diet consumption across classes were examined using the ANOVA (with a normal distribution and homoscedasticity) or the Wilcoxon rank-sum test (without distribution or homoscedasticity) for continuous variables, the chi-square (χ^2^) test for categorical variables, and the Bonferroni correction was used to adjust *p*-value (*P*) to the pairwise comparison between the classes (*P* < 0.007).

Cox proportional hazard regression was applied to test the association between DDS trajectories and the risk of mortality. An unadjusted model and two adjusted models were established. The confounders were identified from univariate analysis (when factors showed a *P* < 0.05). In addition, since the history of chronic disease is usually associated with dietary intake and mortality, it is also considered as a potential confounder in the current analysis. Particularly, in adjusted models, Model 1 adjusted for sociodemographic characteristics, including age at baseline, gender, educational level, region of residence, place of residence, and individual annual income. Model 2 was further adjusted for lifestyle characteristics, including the history of smoking and alcohol consumption, BMI, chronic disease history, physical activity, using hypotensive or hypoglycemic medicine, and energy intake. The proportional hazard assumption of covariates for the Cox regression was tested according to the Schoenfeld residuals test, and the results for DDS trajectory (Supplementary Figure S1) and average DDS (Supplementary Figure S2) showed that all the covariates met the assumption based on a *p*-value threshold of 0.05. The collinearity of the covariates in the adjusted models was examined according to variance inflation factors (VIFs), and the results showed that no collinearity of these covariates existed (all the VIFs were < 5).

Additionally, the Restricted Cubic Spline (RCS) Cox regression was performed to investigate the non-linear relationship between average DDS level and the risk of death, with class 1 (DDS = 3) as the reference and adjusting for the same confounders as Model 2 mentioned above. The regression model with five knots was selected because of the largest coefficient of determination (*R*^2^) and optimism-corrected discrimination index (*D*_xy_) in this model.

All statistical analyses were performed using the R Statistical Software (version 4.1.1, R Development Core Team, Vienna, Austria) (36). *P*-value < 0.05 (two-tailed) was considered statistically significant.

## Results

### Trajectory of DDS changes with age

In total, 6,737 eligible participants were included in the analysis. To explore the trajectory changes of DDS with age, the lowest BIC was reached when the number of classes was four (BIC_4_ = 71920.9), and the number of participants in each class was higher than 5% of the total participants. When only including fixed effects in the trajectory model, the BIC was the lowest and with a better fit, it was therefore selected. No statistical significance was found when adding cubic and quadratic terms in linear models, therefore only a linear model was used.

**Figure 2:**
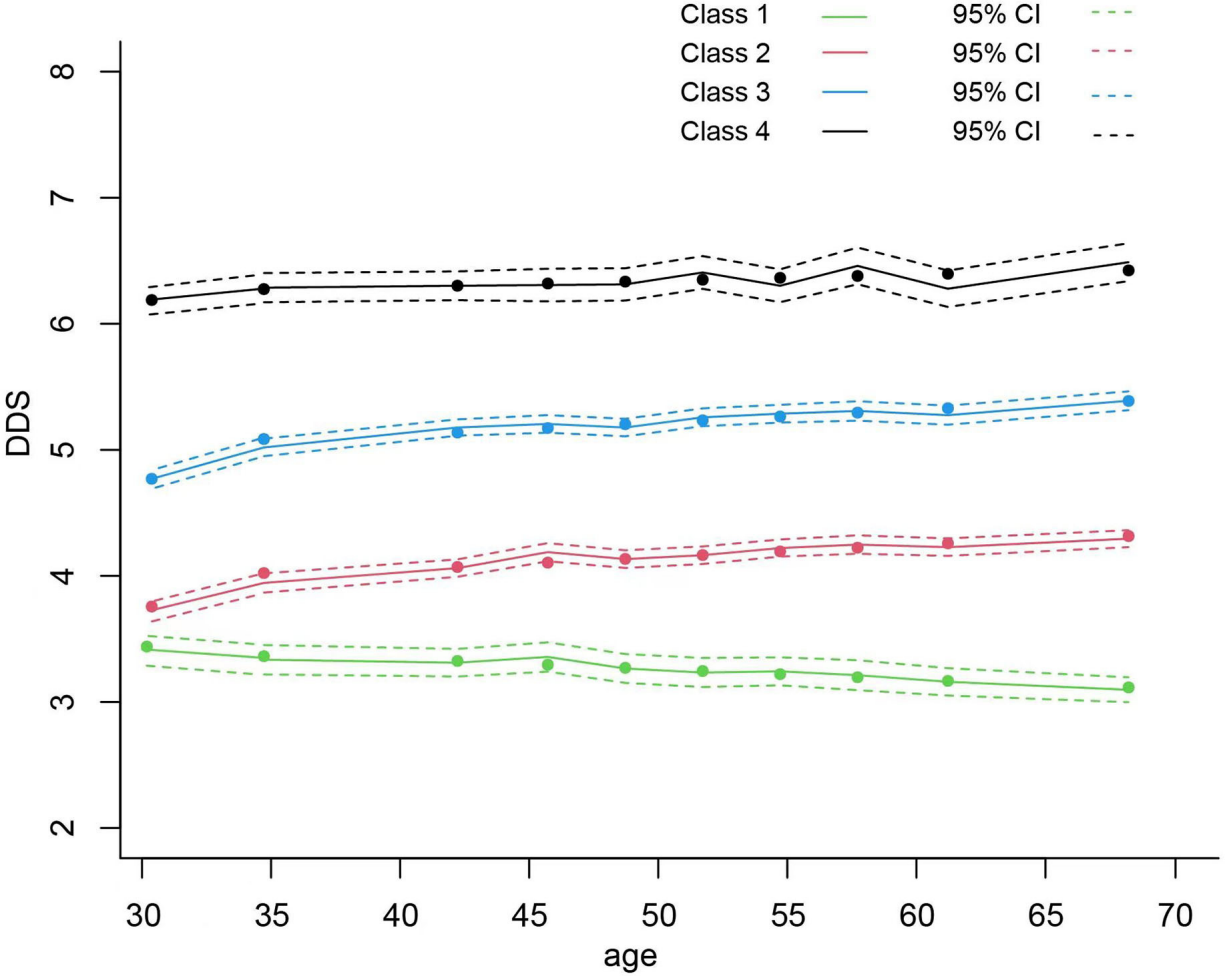
The association between average DDS and mortality hazard ratio using restricted cubic spline analysis with five knots. DDS, dietary diversity score; HR, hazard ratio; CI, confidence interval.

Levels and changes in DDS with age in all four classes are shown in Figure 2. Most of the participants belonged to class 2 and class 3, with 40.6% and 42.2% of participants, respectively. In class 1, the initial DDS was relatively low (intercept = 3.663, *P* < 0.001), and a trend of decreasing with age was observed (β = −0.008, *P* = 0.008). In class 2, the initial DDS was relatively low (intercept = 3.698, *P* < 0.001) and slightly increased with age (β = 0.010, *P* < 0.001). In class 3, the initial DDS was medium (intercept = 4.718, *P* < 0.001) and also slightly increased during the follow-ups (β = 0.010, *P* < 0.001); and in class 4, the initial DDS was the highest among all the classes (intercept = 6.106, *P* < 0.001) and maintained stable (β = 0.005, *P* = 0.147). The mean DDS of classes 1, 2, 3, and 4 during the survey were 3.0, 4.0, 5.2, and 6.7, respectively.

### Baseline characteristics among participants across different classes

Of the studied population, 3,293 (48.9%) were male and 3,444 (51.1%) were female. The median (*P*_25_*, P*_75_) follow-up time was 12.04 (8.94, 13.94) years with 7,6024.36 person-years. During the follow-ups, 326 deaths occurred. Specifically, 64, 151, 106, and 5 participants died in class 1, class 2, class 3, and class 4, respectively.

Table 1 shows the baseline characteristics across classes. The ages of participants at baseline were significantly different between classes, with a decreasing trend from class 1 (median = 48.0) to class 4 (median = 42.0). Compared to class 1, class 4 was more likely to have a higher education level, annual individual income, BMI, and energy intake, and class 4 tended to have less physical activity and live in urban areas.

**Table 1:** Characteristics of participants across different classes.

	**Class 1**	**Class 2**	**Class 3**	**Class 4**	***P***
	***N* = 614**	***N* = 2735**	***N* = 2841**	***N* = 547**	
Age at baseline, years, *M* (*P_25_, P_75_*)	48.0 (41.0, 54.0)^abc^	44.0 (37.0, 50.0)^ade^	42.0 (35.0, 49.0)^bd^	42.0 (35.0, 48.0)^c^	< 0.001
**Gender**, ***n*** **(%)**					0.036
Male	285 (46.4)	1341 (49.0)	1426 (50.2)	241 (44.1)
Female	329 (53.6)	1394 (51.0)	1415 (49.8)	306 (55.9)
**Level of education**, ***n*** **(%)**					< 0.001
Junior high school or below	571 (94.4)^abc^	2308 (85.1)^ade^	1991 (70.6)^bdf^	246 (45.2)^cef^
Senior high school or above	34 (5.62)	403 (14.9)	830 (29.4)	298 (54.8)
**Region of residence**, ***n*** **(%)**					< 0.001
Eastern city	100 (16.3)^abc^	724 (26.5)^ade^	1122 (39.5)^bdf^	296 (54.1)^cef^
Central city	367 (59.8)	1134 (41.5)	1259 (44.3)	220 (40.2)
Western city	147 (23.9)	877 (32.1)	460 (16.2)	31 (5.67)
**Place of residence**					< 0.001
Urban area	91 (14.8)^ab^	528 (19.3)^cd^	1141 (40.2)^ace^	417 (76.2)^bde^
Rural area	523 (85.2)	2207 (80.7)	1700 (59.8)	130 (23.8)
Annual individual income, yuan, *M* (*P_25_, P_75_*)	3221 (1250, 5567)^abc^	5217 (2168, 10737)^ade^	10317 (4934, 17294)^bdf^	15789 (9642, 23339)^cef^	< 0.001
**Smoking history**, ***n*** **(%)**					0.004
Never	351 (57.2)^a^	1569 (57.5)^b^	1681 (59.3)^c^	358 (56.5)^abc^
Yes	263 (42.8)	1162 (42.5)	1153 (40.7)	188 (34.4)
**Alcohol history**, ***n*** **(%)**					< 0.001
Never	349 (56.8)^ab^	1435 (52.5)^c^	1413 (49.8)^a^	246 (45.0)^bc^
Yes	265 (43.2)	1300 (47.5)	1423 (50.2)	301 (55.0)
BMI, kg/m^2^, *M* (*P_25_, P_75_*)	22.0 (20.4, 24.8)^ab^	22.8 (20.9, 25.2)^cd^	23.6 (21.4, 25.8)^ac^	23.3 (21.4, 25.6)^bd^	< 0.001
**Chronic disease history**, ***n*** **(%)**					0.069
No	414 (67.4)	1910 (69.8)	1891 (66.6)	369 (67.5)
Yes	200 (32.6)	825 (30.2)	950 (33.4)	178 (32.5)
**Hypotensive or hypoglycemic medicine taking**, ***n*** **(%)**					0.009
No	506 (82.4)	2254 (82.4)^a^	2249 (79.2)^a^	433 (79.2)
Yes	108 (17.6)	481 (17.6)	592 (20.8)	114 (20.8)
Physical activity, MET hour/week, *M* (*P_25_, P_75_*)	2691 (664, 4599)^abc^	2553 (594, 5083)^ade^	1243 (378, 3275)^bdf^	1003 (475, 1942)^cef^	< 0.001
Energy intake, kcal/day, *M* (*P_25_, P_75_*)	2013 (1663, 2359)^abc^	2120 (1825, 2474)^a^	2157 (1870, 2491)^b^	2177 (1904, 2505)^c^	< 0.001
Dietary diversity score, *M* (*P_25_, P_75_*)	3.0 (2.5, 3.2)^abc^	4.0 (3.8, 4.3)^ade^	5.2 (5.0, 5.8)^bdf^	6.7 (6.3, 7.0)^cef^	< 0.001
Number of death	64	151	106	5	< 0.001
Incidence (number of deaths/1000 person-years)	8.4^abc^	4.8^ade^	3.4^bdf^	0.9^cef^	< 0.001

The differences in characteristics across classes were examined by the Wilcoxon rank-sum test for continuous variables, and the chi-square (χ^2^) test for categorical variables. Superscript letters (abcdef) denoted statistically significant pairwise comparisons (following Bonferroni correction of P < 0.007). BMI, body mass index; M, median; P_25_, 25th percentile; P_75_, 75th percentile.

### Association between the trajectory of DDS changes, average DDS, and all-cause mortality

A total of 326 deaths occurred during the follow-ups. The highest and lowest incidences of death were observed in class 1 and class 4, with 8.4 deaths/1,000 person-years and 0.9 deaths/1,000 person-years, respectively. Results of Cox proportional hazard regression showed that significant differences in mortality among all the classes were observed in the unadjusted model. The most substantial difference was found in class 4, with 86% of lower mortality (*HR*: 0.14; 95% *CI*: 0.06–0.35) compared to class 1. When adjusted for sociodemographic characteristics (model 1), only class 4 showed significantly lower mortality compared with other classes. After adjusting for both sociodemographic characteristics and lifestyle characteristics (model 2), the results were similar: class 4 was associated with 71% (*HR*:0.29; 95% *CI*: 0.10–0.83), 68% (*HR*:0.32; 95% *CI*: 0.11–0.89), and 66% (*HR*:0.34; 95% *CI*: 0.12–0.94) of reduced risk of mortality compared to class 1, 2, and 3, respectively. However, no significant inter-class differences were observed between the other three classes (Table 2).

**Table 2:** Associations of DDS trajectory and average DDS with mortality.

**Comparable group vs. Reference**	**Unadjusted Model**	**Model 1**	**Model 2**
Class 2 vs. Class 1	0.71 (0.52, 0.96)*	0.91 (0.67, 1.24)	0.90 (0.65, 1.26)
Class 3 vs. Class 1	0.53 (0.39, 0.73)***	0.85 (0.60, 1.20)	0.84 (0.58, 1.23)
Class 3 vs. Class 2	0.75 (0.58, 0.96)*	0.94 (0.72, 1.22)	0.93 (0.71, 1.24)
Class 4 vs. Class 1	0.14 (0.06, 0.35)***	0.33 (0.13, 0.86)*	0.29 (0.10, 0.83)*
Class 4 vs. Class 2	0.20 (0.08, 0.49)***	0.36 (0.15, 0.92)*	0.32 (0.11, 0.89)*
Class 4 vs. Class 3	0.27 (0.11, 0.66)**	0.39 (0.16, 0.97)*	0.34 (0.12, 0.94)*
Hazard ratio of average DDS and mortality	0.69 (0.62, 0.76)***	0.79 (0.70, 0.89)***	0.78 (0.69, 0.89)***

Cox proportional hazard regression was used to test the associations between DDS trajectory and average DDS with mortality. Model 1 adjusted for age at baseline, gender, level of education, region of residence, place of residence, and individual annual income. Model 2 based on Model 1 further adjusted for history of smoking and alcohol consumption, BMI, history of chronic disease, physical activity, hypotensive or hypoglycemic medicine, and energy intake. *P < 0.05, **P < 0.01, ***P < 0.001. DDS, dietary diversity score.

Furthermore, we calculated the average DDS among participants during the follow-ups and found that after adjusting for both sociodemographic characteristics and lifestyle characteristics (model 2), a higher average DDS was significantly associated with a reduced risk of mortality. Each point of increase in DDS corresponded to a 22% reduced risk of mortality in the whole research population (*HR*:0.78; 95% *CI*: 0.69–0.89).

**Figure 3:**
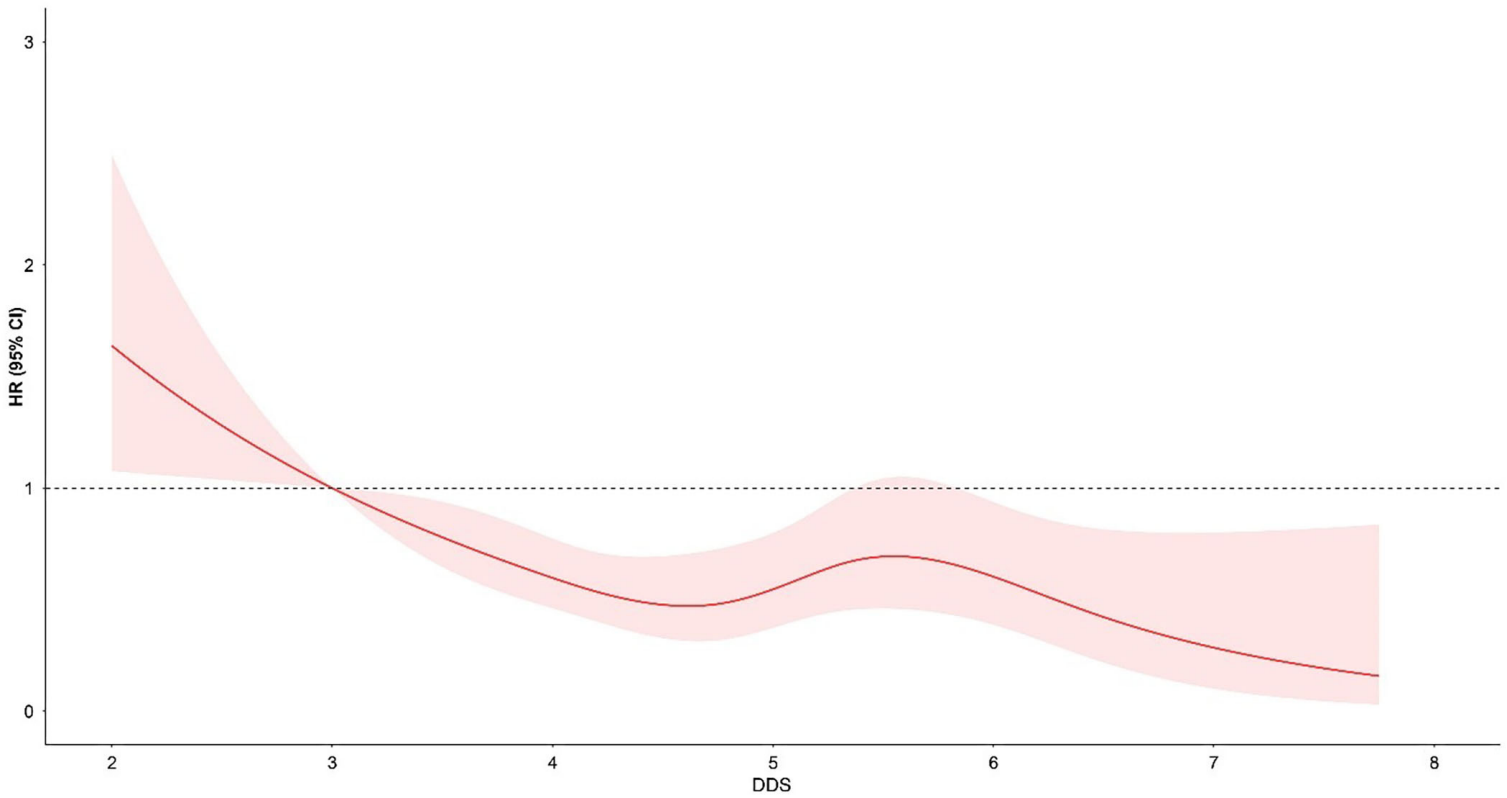
Levels and trajectories of DDS change with the age of participants per class. DDS, dietary diversity score.

Additionally, the results of the cubic spline curve showed a negatively non-linear association between all-cause mortality and average DDS during the follow-ups. Compared with DDS = 3, DDS < 3 was associated with a higher risk of mortality, and DDS > 3 was associated with an increased risk of mortality, despite a slight fluctuation between DDS = 5 and DDS = 6 (Figure 3).

The hazard ratio was indicated by the solid line, and 95% confidence intervals were represented by the shaded area. The reference point was DDS = 3, adjusted for age at baseline, gender, levels of education, regions of residence, places of residence, and individual annual income, as well as the history of smoking and alcohol consumption, BMI, history of chronic disease, physical activity, hypotensive or hypoglycemic medicine, and energy intake.

## Discussion

To the best of our knowledge, this is the first study investigating a long-term dynamic change of DDS and its association with mortality in the Chinese population. Our study revealed that reaching and maintaining a higher dietary diversity was significantly associated with lower mortality in the Chinese population.

### Average DDS and mortality

DDSs have been widely accepted as relatively uncomplicated and promising tools to quantify dietary diversity on a large scale (37). The development of DDSs could be based on different measurements, such as the number of individual food items consumed (38), the number of different food groups consumed with the standard weight for each food group (39), as well as the number of food groups consumed that are allocated with different weights according to dietary guidelines (40). Types of dietary assessment also varied. The most commonly used method is 24-h recall, followed by a food frequency questionnaire, food record, and diet history (41). In our study, we used the food-group-based measurement to calculate DDS, which is also the most frequently used method of developing DDSs (41). And food intakes of individuals were calculated using three days of 24-h recall and validated with weighed food inventory per household, which further improved the accuracy of dietary intake data.

Generally, dietary diversity can be an indicator of nutritional adequacy and food accessibility, thus reflecting the potential risk of malnutrition in elderlies, younger adults, and children (21, 42–44). The Chinese dietary guidelines 2022 recommend that people should eat more than 12 kinds of food every day, and more than 25 kinds of food every week (30). Since the DDS used in our study was developed based on the Chinese dietary guidelines, a higher DDS could reflect adherence to the dietary guidelines. However, in some cases, higher DDS may also lead to excessive energy intake, thus leading to overweight or obesity (42). This could be due to an unbalanced diet, such as consuming more varieties of sweets, snacks, and carbohydrates, especially in upper-middle-income economies (21, 45). Nevertheless, a recent systematic scoping review indicated that higher dietary diversity was positively associated with benefits for various health outcomes such as non-communicable diseases, biomarkers of nutritional status, and mental health as well as cognitive functions among both adolescents and adults (41). However, studies on the relation between dietary diversity and all-cause mortality, especially in developing countries, are still scant. Nevertheless, this review also pointed out that the mixed or null associations between DDS and health outcomes sometimes were shown. Furthermore, the follow-up time of most previous studies was too short to reveal the relation between dietary diversity and health outcomes (41).

In our study, we found that a high level of average DDS related to significantly lower all-cause mortality. Furthermore, each point of increase in average DDS was associated with a 23% reduced risk of mortality among the middle-aged and elderly population. The fitted curve of association between DDS and mortality hazard ratio showed that DDS of lower than 3 was significantly associated with an increased all-cause mortality rate, and DDS of higher than three was associated with a decreased mortality rate. These results are in line with another study, which concluded that among older adults, a higher overall DDS was associated with a 30% decreased risk of mortality (46). The higher decrease in risk of mortality compared with our study might be due to the older age of the subjects (65 or older at baseline). The possible explanation for the inverse association between DDS and mortality could be the fact that DDS could reflect micronutrient adequacy (42). Also, a diverse diet could increase gut microbiota diversity, which was associated with nutritional status, inflammatory disorders, and co-morbidity (47). In addition, DDS lower than three is also regarded as an indicator of malnutrition in Ethiopian children, which predicted to be underweight (48). These results indicated that DDS of lower than 3 might be an important cut-off value to reflect the poor dietary quality and to predict poor health status among not only children but also middle-aged and elderly populations.

It is also noteworthy that people with a lower DDS were more likely to live in rural areas, have lower income, a lower education level, and a higher physical activity level. This could be due to better accessibility to varieties of food in urban areas than in rural areas (49). In addition, less education of them might lead to a lack of nutrition knowledge, engagement in low-paid physical labor, and significantly lower energy intake. Therefore, increasing and maintaining the nutritional status of this population for the long term is crucial. Policymakers should pay more attention to increasing the diversity of food supply in rural areas, promoting the accessibility of different kinds of foods, and providing financial support to people with low income, thereby improving their wellbeing and decreasing the mortality rate of the vulnerable population in China.

### Longitudinal changes of DDS and their associations with mortality

Besides the health influence of DDS, the changes of DDS along with age may also bring a significant impact on health. In the aging process, people usually experience changes in multiple physical, mental, or lifestyle factors that may decrease their dietary diversity. Physically, sensory function decline such as loss of taste and smell may reduce total food intake, thus leading to worsened nutritional state (50). Moreover, the lower chewing ability of elderly people is also associated with food intake insufficiency (15). The lifestyle of elderly people such as eating alone is related to depressive mood and thus decreases their appetite and dietary diversity (16). The financial disadvantage is another hurdle that may limit the dietary diversity of the elderlies, as a study reported that a diverse diet was nearly one-fifth more expensive than an undiversified diet (6, 14).

With the LCTM approach, the dynamic changes of DDS along with aging in the Chinese population were first addressed. Interestingly, DDS did not show a trend of decrease in most populations, although it was prevalent in elderly people (17). In our study, only people in class 1 with the lowest average DDS and the highest median age of baseline showed decreased DDS over time. In classes with relatively low to medium DDS (classes 2 and 3), increases in DDS over the years were observed. However, after adjusting for sociodemographic and lifestyle characteristics, no significant positive effects on mortality were detected compared with class 1. Given that more than 90% of the studied participants were in classes 1, 2, and 3, it can be beneficial for them to increase DDS to a higher level.

A previous longitudinal study showed that elderly people with lower DDS caused by poor appetite had a higher risk of all-cause mortality (51). Participants of this study with a low DDS scantly consumed multiple kinds of food, including vegetables and fruits, fish and other seafood, meat, and eggs, which might result in their lower intakes of total energy, protein, vitamin B, iron, and phosphate. Not only malnutrition is widely accepted as a health concern and significantly shortens the life expectancy of older adults, but also low consumption of varieties of food and nutrients caused by low appetite could be a reason for the higher mortality rate (52). Inadequate protein-rich food consumption, together with a lower DDS, was suggested to be associated with a higher all-cause mortality rate in the elderly (53). Also, because of the diminished sense of taste, together with the monotonous diet of people with low DDS, elderly people tend to eat food with strong flavors, such as pickled vegetables with high salt and sodium content and sweet food containing large amounts of sugar (51, 54). High sodium intake may attribute to additional health risks of chronic diseases, such as hypertension, and excessive sugar consumption is associated with a higher risk of type 2 diabetes and obesity (55). Malnutrition and the higher risk of chronic diseases may both increase the mortality rate of elderly people. Nevertheless, it should be noted that an extreme increase or decrease in DDS was associated with a higher risk of mortality (10). The possible explanation could be that dramatic change in lifestyle could be because of unstable family care, irregular physical exercise, or the diagnosis of chronic diseases.

China is transitioning into an aging society. Since aging is associated with various chronic disorders, more healthcare and medical support for elderly people aggravate the burden on their families and society. Diet is a modifiable factor that is relatively easy and inexpensive to change. Thus, promoting a higher dietary diversity can be a promising way to decrease the mortality rate and prolong health spans in elderly people. The CDG-2022 suggests that elderly people should consume at least 12 kinds of foods every day, and increase their appetite with varieties of methods, such as changing cooking recipes and eating with families and friends (30). Yet, policymakers should place more emphasis on promoting a higher dietary diversity by multiple means such as increasing the accessibility and affordability of a wider range of foods.

### Strengths and weaknesses

This study has several strengths. First, the data we used was based on a large sample cohort with a long follow-up time of more than 10 years. Second, we performed a new and reasonable method for trajectory analysis, which could reflect the dynamic changes of DDS over the years. However, limitations in this study warrant careful consideration. Although we have adjusted for many potential confounders, there could still be unmeasured or residual confounders that could not be completely ruled out. In addition, we have only investigated the association between DDS changes and all-cause mortality, because the cause-specific mortality rates were not available in the CHNS. Therefore, the association between DDS and cause-specific mortality is still needed to be explored to understand the important role that DDS played in human health. Also, specific food groups involved in DDS, such as plant- or animal-based food groups, and their associations with mortality should be further investigated.

## Conclusion

Reaching and maintaining a higher DDS among middle-aged and the elderly is significantly associated with a decreased risk of all-cause mortality. These findings demonstrate the significance of promoting and maintaining a higher DDS over middle to late adulthood. Future studies should focus on not only DDS and its influencing factors but also the change in DDS over the life course to further clarify the health implications of maintaining a high dietary diversity, such as malnutrition, metabolic diseases and other non-communicable diseases, and mental health.

## Data availability statement

Publicly available datasets were analyzed in this study. This data can be found at: https://www.cpc.unc.edu/projects/china.

## Ethics statement

The studies involving human participants were reviewed and approved by the Institutional Review Boards at the University of North Carolina (Chapel Hill, NC, the US); the National Institute of Nutrition and Food Safety (Chinese Center for Disease Control and Prevention); Institution Review Board of Tsinghua University. The patients/participants provided their written informed consent to participate in this study.

## Author contributions

AZ designed the study, edited, and proofread the final manuscript, has full access to all data used in this study, and has taken responsibility for the integrity of the data and the accuracy of the data analysis. XN performed the statistical analyses. XQ wrote the manuscript, interpreted the results, and composed the discussion. JY, HY, and AC supported literature searching and data analysis. All authors have read and approved the final manuscript.

## Funding

Data used in this study are from the China Health and Nutrition Survey (CHNS) project, which was funded by several organizations. Major funding for the survey and data dissemination from 1991 to 2004 came from the National Institutes of Health (NIH) (P01-HD28076 and HD30880). Additional funding was from the NIH (HD39183), the Carolina Population Center, the Ford Foundation, the National Science Foundation (INT-9215399), the National Institute of Nutrition and Food Safety, and the Chinese Center for Disease Control and Prevention.

## Conflict of interest

The authors declare that the research was conducted in the absence of any commercial or financial relationships that could be construed as a potential conflict of interest.

## Publisher's note

All claims expressed in this article are solely those of the authors and do not necessarily represent those of their affiliated organizations, or those of the publisher, the editors and the reviewers. Any product that may be evaluated in this article, or claim that may be made by its manufacturer, is not guaranteed or endorsed by the publisher.

## Supplementary material

Supplementary Figure S1 Proportional hazard assumption of covariates for the Cox regression of dietary diversity score trajectory and mortality according to the Schoenfeld residuals test, and the results showed that all the covariates met the assumption based on a *p*-value threshold of 0.05 (*P* = 0.08).

Supplementary Figure S2 Proportional hazard assumption of covariates for the Cox regression of average dietary diversity score and mortality according to the Schoenfeld residuals test, and the results showed that all the covariates met the assumption based on a *p*-value threshold of 0.05 (*P* = 0.14).

## References

[1] U.S. Department of Agriculture and U.S. Department of Health and Human Services. Dietary Guidelines for Americans, 2020–2025. 9th ed. (2020). Available online at: https://www.dietaryguidelines.gov/resources/2020-2025-dietary-guidelines-online-materials

[2] National Health and Medical Research Council. Australian Dietary Guidelines Summary. Canberra: National Health and Medical Research Council (2013).

[3] Wang SS Lay S Yu HN Shen SR. Dietary guidelines for chinese residents (2016): comments and comparisons. J Zhejiang Univ Sci B. (2016) 17:649–56. 10.1631/jzus.B1600341 27604857 PMC5018612

[4] Kennedy GB Dop T. Guidelines for Measuring Household and Individual Dietary Diversity. Rome: Food and Agriculture Organization of the United Nations. (2011).

[5] Abris GP Provido SMP Hong S Yu SH Lee CB Lee JE. Association between dietary diversity and obesity in the Filipino Women's Diet and Health Study (FiLWHEL): a cross-sectional study. PLoS ONE. (2018) 13:e0206490. 10.1371/journal.pone.0206490 30383830 PMC6211689

[6] Conklin AI Monsivais P Khaw KT Wareham NJ Forouhi NG. Dietary diversity, diet cost, and incidence of type 2 diabetes in the united kingdom: a prospective cohort study. PLoS Med. (2016) 13:e1002085. 10.1371/journal.pmed.1002085 27433799 PMC4951147

[7] Otsuka R Nishita Y Tange C Tomida M Kato Y Nakamoto M. Dietary diversity decreases the risk of cognitive decline among Japanese older adults: dietary diversity and cognitive decline. Geriatr Gerontol Int. (2017) 17:937–44. 10.1111/ggi.12817 27380888

[8] Zhang J Zhao A Wu W Yang C Ren Z Wang M. Dietary diversity is associated with memory status in chinese adults: a prospective study. Front Aging Neurosci. (2020) 12:580760. 10.3389/fnagi.2020.580760 33117146 PMC7494158

[9] Zheng J Zhou R Li F Chen L Wu K Huang J. Association between dietary diversity and cognitive impairment among the oldest-old: findings from a nationwide cohort study. Clin Nutr. (2021) 40:1452–62. 10.1016/j.clnu.2021.02.041 33740515

[10] Liu D Zhang XR Li ZH Zhang YJ Lv YB Wang ZH. Association of dietary diversity changes and mortality among older people: a prospective cohort study. Clin Nutr. (2021) 40:2620–29. 10.1016/j.clnu.2021.04.012 33933728

[11] Miyamoto K Kawase F Imai T Sezaki A Shimokata H. Dietary diversity and healthy life expectancy-an international comparative study. Eur J Clin Nutr. (2019) 73:395–400. 10.1038/s41430-018-0270-3 30104730

[12] Kobayashi M Sasazuki S Shimazu T Sawada N Yamaji T Iwasaki M. Association of dietary diversity with total mortality and major causes of mortality in the Japanese population: JPHC study. Eur J Clin Nutr. (2020) 74:54–66. 10.1038/s41430-019-0416-y 30890778

[13] Morley JE. Decreased food intake with aging. J Gerontol A Biol Sci Med Sci. (2001) 56:81–8. 10.1093/gerona/56.suppl_2.81 11730241

[14] Lo YT Chang YH Lee MS Wahlqvist ML. Dietary diversity and food expenditure as indicators of food security in older Taiwanese. Appetite. (2012) 58:180–7. 10.1016/j.appet.2011.09.023 22001748

[15] Kimura Y Ogawa H Yoshihara A Yamaga T Takiguchi T Wada T. Evaluation of chewing ability and its relationship with activities of daily living, depression, cognitive status and food intake in the community-dwelling elderly. Geriatr Gerontol Int. (2013) 13:718–25. 10.1111/ggi.12006 23279752

[16] KimuraYWadaTOkumiyaKIshimotoYFukutomiEKasaharaY. Eating alone among community-dwelling Japanese elderly: association with depression and food diversity. J Nutr Health Aging. (2012) 16:728–31. 10.1007/s12603-012-0067-323076516

[17] OtsukaRNishitaYTangeCTomidaMKatoYImaiT. Age-related 12-year changes in dietary diversity and food intakes among community-dwelling Japanese aged 40 to 79 years. J Nutr Health Aging. (2018) 22:594–600. 10.1007/s12603-018-0999-329717759

[18] Chang X DeFries RS Liu L Davis K. Understanding dietary and staple food transitions in China from multiple scales. PLoS ONE. (2018) 13:e0195775. 10.1371/journal.pone.0195775 29689066 PMC5915834

[19] Lu J Liu Q. Four decades of studies on population aging in China. China Population Dev Stud. (2019) 3:24–36. 10.1007/s42379-019-00027-4

[20] Huang Q Wang L Jiang H Wang H Zhang B Zhang J. Intra-individual double burden of malnutrition among adults in china: evidence from the China health and nutrition survey 2015. Nutrients. (2020) 12:2811. 10.3390/nu12092811 32937736 PMC7551383

[21] Foote JA Murphy SP Wilkens LR Basiotis PP Carlson A. Dietary variety increases the probability of nutrient adequacy among adults. J Nutr. (2004) 134:1779–85. 10.1093/jn/134.7.1779 15226469

[22] Liu Z Zhao L Man Q Wang J Zhao W Zhang J. Dietary micronutrients intake status among Chinese elderly people living at home: data from CNNHS 2010–2012. Nutrients. (2019) 11:1787. 10.3390/nu11081787 31382399 PMC6722721

[23] Zhang B Zhai FY Du SF Popkin BM. The China health and nutrition survey, 1989–2011. Obes Rev. (2014) 15:2–7. 10.1111/obr.12119 24341753 PMC3869031

[24] Zhai F Guo X Popkin BM Ma L Wang Q Shuigao WY. Evaluation of the 24-hour individual recall method in China. Food Nutr Bullet. (1996) 17:1–7. 10.1177/156482659601700209

[25] Fang A Li K Guo M He J Li H Shen X. Long-term low intake of dietary calcium and fracture risk in older adults with plant-based diet: a longitudinal study from the China health and nutrition survey. J Bone Miner Res. (2016) 31:2016–23. 10.1002/jbmr.2874 27208802

[26] Wang G. China Food Composition 1991. Beijing: Peking University Medical Press (1991).

[27] Yang Y Wang G Pan X. China Food Composition 2002. Beijing: Peking University Medical Press (2002).

[28] Yang Y Wang G Pan X. China Food Composition 2004. Beijing: Peking University Medical Press (2005).

[29] Zhai F Du S Wang Z Zhang J Du W Popkin BM. Dynamics of the Chinese diet and the role of urbanicity, 1991–2011. Obesity Rev. (2014) 15:16–26. 10.1111/obr.12124 24341755 PMC3868998

[30] Chinese Nutrition Society,. Chinese Dietary Guidelines. People's Medical Publishing House. (2022). Available online at: http://dg.cnsoc.org/article/04/J4-AsD_DR3OLQMnHG0-jZA.html

[31] Zhang J Liang D Zhao A. Dietary diversity and the risk of fracture in adults: a prospective study. Nutrients. (2020) 12:3655. 10.3390/nu12123655 33261013 PMC7761242

[32] Salehi-Abargouei A Akbari F Bellissimo N Azadbakht L. Dietary diversity score and obesity: a systematic review and meta-analysis of observational studies. Eur J Clin Nutr. (2016) 70:1–9. 10.1038/ejcn.2015.118 26220567

[33] Lennon H Kelly S Sperrin M Buchan I Cross AJ Leitzmann M. Framework to construct and interpret latent class trajectory modelling. BMJ Open. (2018) 8:e020683. 10.1136/bmjopen-2017-020683 29982203 PMC6042544

[34] Twisk J Hoekstra T. Classifying developmental trajectories over time should be done with great caution: a comparison between methods. J Clin Epidemiol. (2012) 65:1078–87. 10.1016/j.jclinepi.2012.04.010 22818946

[35] Schwandt A Hermann JM Rosenbauer J Boettcher C Dunstheimer D Grulich-Henn J. Longitudinal trajectories of metabolic control from childhood to young adulthood in type 1 diabetes from a large German/Austrian registry: a group-based modeling approach. Diab Care. (2017) 40:309–16. 10.2337/dc16-1625 28007778

[36] R Core Team. R: A language and environment for statistical computing. R Foundation for Statistical Computing. Vienna (2021).

[37] Ruel MT. Operationalizing dietary diversity: a review of measurement issues and research priorities. J Nutr. (2003) 133:11S−26S. 10.1093/jn/133.11.3911S 14672290

[38] Roberts SB Hajduk CL Howarth NC Russell R McCrory MA. Dietary variety predicts low body mass index and inadequate macronutrient and micronutrient intakes in community-dwelling older adults. J Gerontol A Biol Sci Med Sci. (2005) 60:613–21. 10.1093/gerona/60.5.613 15972614

[39] Mirmiran P Azadbakht L Esmaillzadeh A Azizi F. Dietary diversity score in adolescents – a good indicator of the nutritional adequacy of diets: Tehran lipid and glucose study. Asia Pac J Clin Nutr. (2004) 13:56–60. 15003915

[40] Drescher LS Thiele S Mensink GB. A new index to measure healthy food diversity better reflects a healthy diet than traditional measures. J Nutr. (2007) 137:647–51. 10.1093/jn/137.3.647 17311954

[41] Verger EO Le Port A Borderon A Bourbon G Moursi M Savy M. Dietary diversity indicators and their associations with dietary adequacy and health outcomes: a systematic scoping review. Adv Nutr. (2021) 12:1659–72. 10.1093/advances/nmab009 33684194 PMC8483968

[42] Zhao W Yu K Tan S Zheng Y Zhao A Wang P. Dietary diversity scores: an indicator of micronutrient inadequacy instead of obesity for Chinese children. BMC Pub Health. (2017) 17:440. 10.1186/s12889-017-4381-x 28499361 PMC5429576

[43] Oldewage-Theron WH Kruger R. Food variety and dietary diversity as indicators of the dietary adequacy and health status of an elderly population in Sharpeville, South Africa. J Nutr Elder. (2008) 27:101–33. 10.1080/01639360802060140 18928193

[44] Zhao A Li Z Ke Y Huo S Ma Y Zhang Y. Dietary Diversity among Chinese Residents during the COVID-19 outbreak and its associated factors. Nutrients. (2020) 12:1699. 10.3390/nu12061699 32517210 PMC7352896

[45] Zhang Q Chen X Liu Z Varma DS Wan R Zhao S. Diet diversity and nutritional status among adults in southwest China. PLoS ONE. (2017) 12:e0172406. 10.1371/journal.pone.0172406 28231308 PMC5322886

[46] Tao L Xie Z Huang T. Dietary diversity and all-cause mortality among Chinese adults aged 65 or older: a community-based cohort study. Asia Pac J Clin Nutr. (2020) 29:152–60. 32229454 10.6133/apjcn.202003_29(1).0020

[47] Claesson MJ Jeffery IB Fitzgerald GF Deane J O'Connor M Harnedy N. Gut microbiota composition correlates with diet and health in the elderly. Nature. (2012) 488:178–84. 10.1038/nature11319 22797518

[48] Tosheno D Mehretie Adinew Y Thangavel T Bitew Workie S. Risk factors of underweight in children aged 6–59 months in Ethiopia. J Nutr Metab. (2017) 2017:6368746. 10.1155/2017/6368746 29259827 PMC5702944

[49] Liu J Shively GE Binkley JK. Access to variety contributes to dietary diversity in China. Food Policy. (2014) 49:323–31. 10.1016/j.foodpol.2014.09.007

[50] Mathieu ME Reid RE King NA. Sensory profile of adults with reduced food intake and the potential roles of nutrition and physical activity interventions. Adv Nutr. (2019) 10:1120–5. 10.1093/advances/nmz044 31121014 PMC6855938

[51] Huang YC Wahlqvist ML Lee MS. Appetite predicts mortality in free-living older adults in association with dietary diversity. A NAHSIT cohort study. Appetite. (2014) 83:89–96. 10.1016/j.appet.2014.08.017 25131903

[52] Norman K Haß U Pirlich M. Malnutrition in older adults-recent advances and remaining challenges. Nutrients. (2021) 13:2764. 10.3390/nu13082764 34444924 PMC8399049

[53] Lv Y Kraus VB Gao X Yin Z Zhou J Mao C. Higher dietary diversity scores and protein-rich food consumption were associated with lower risk of all-cause mortality in the oldest old. Clin Nutr. (2020) 39:2246–54. 10.1016/j.clnu.2019.10.012 31685303 PMC7182467

[54] Sergi G Bano G Pizzato S Veronese N Manzato E. Taste loss in the elderly: Possible implications for dietary habits. Crit Rev Food Sci Nutr. (2017) 57:3684–9. 10.1080/10408398.2016.1160208 27129026

[55] Macdonald IA. A review of recent evidence relating to sugars, insulin resistance and diabetes. Eur J Nutr. (2016) 55:17–23. 10.1007/s00394-016-1340-8 27882410 PMC5174139

