# Supplementary figures and images for: Reaching and maintaining higher dietary diversity is associated with decreased risk of all-cause mortality: A longitudinal study from the China Health and Nutrition Survey
Source: Front Nutr. 2022 Sep 26;9:947290. doi: 10.3389/fnut.2022.947290 (PMC9549334; doi:10.3389/fnut.2022.947290)

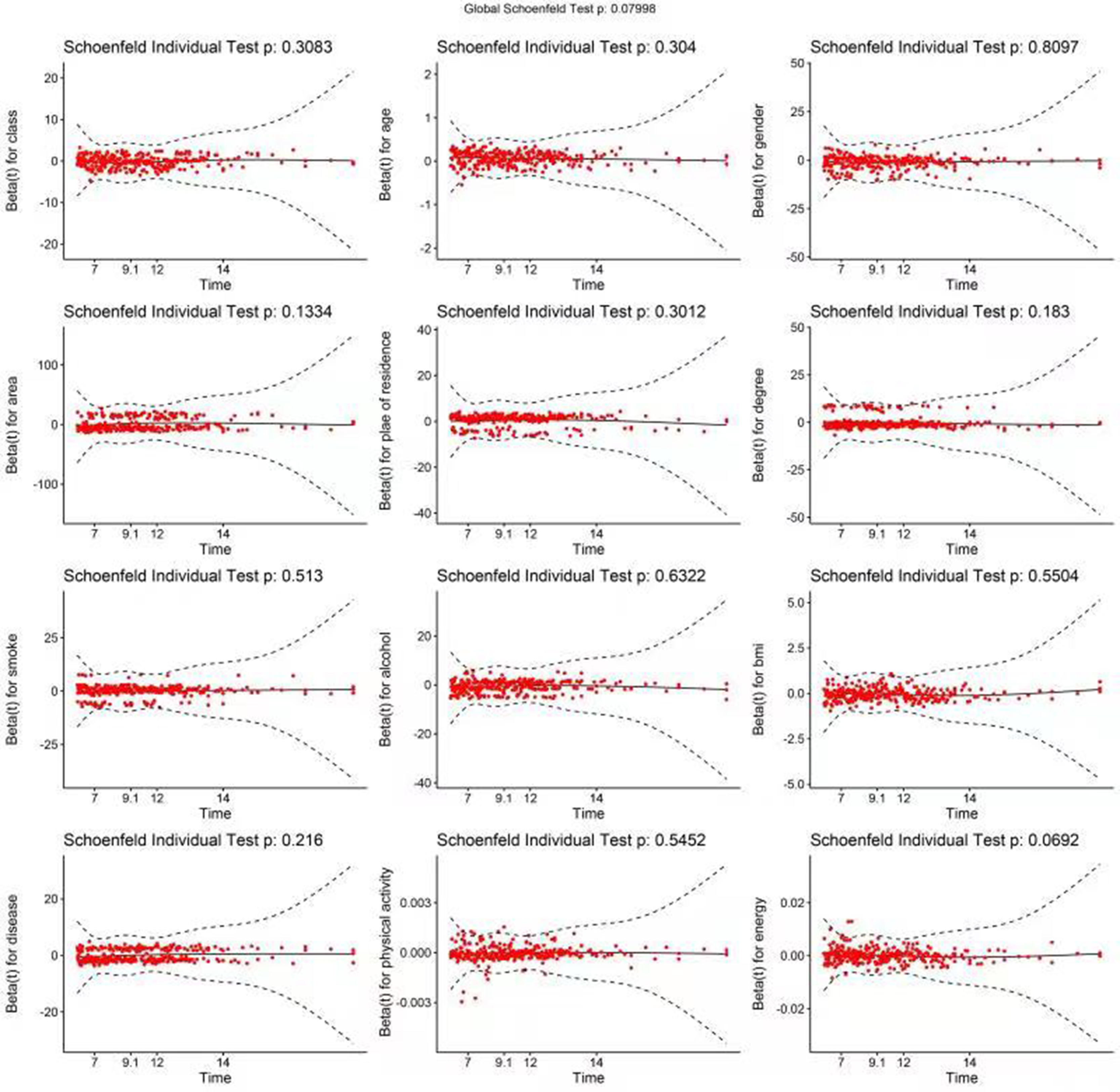

Supplement: Supplementary Figure S1 — Proportional hazard assumption of covariates for the Cox regression of dietary diversity score trajectory and mortality according to the Schoenfeld residuals test, and the results showed that all the covariates met the assumption based on a p-value threshold of 0.05 (P = 0.08). [file Image_1.jpg]

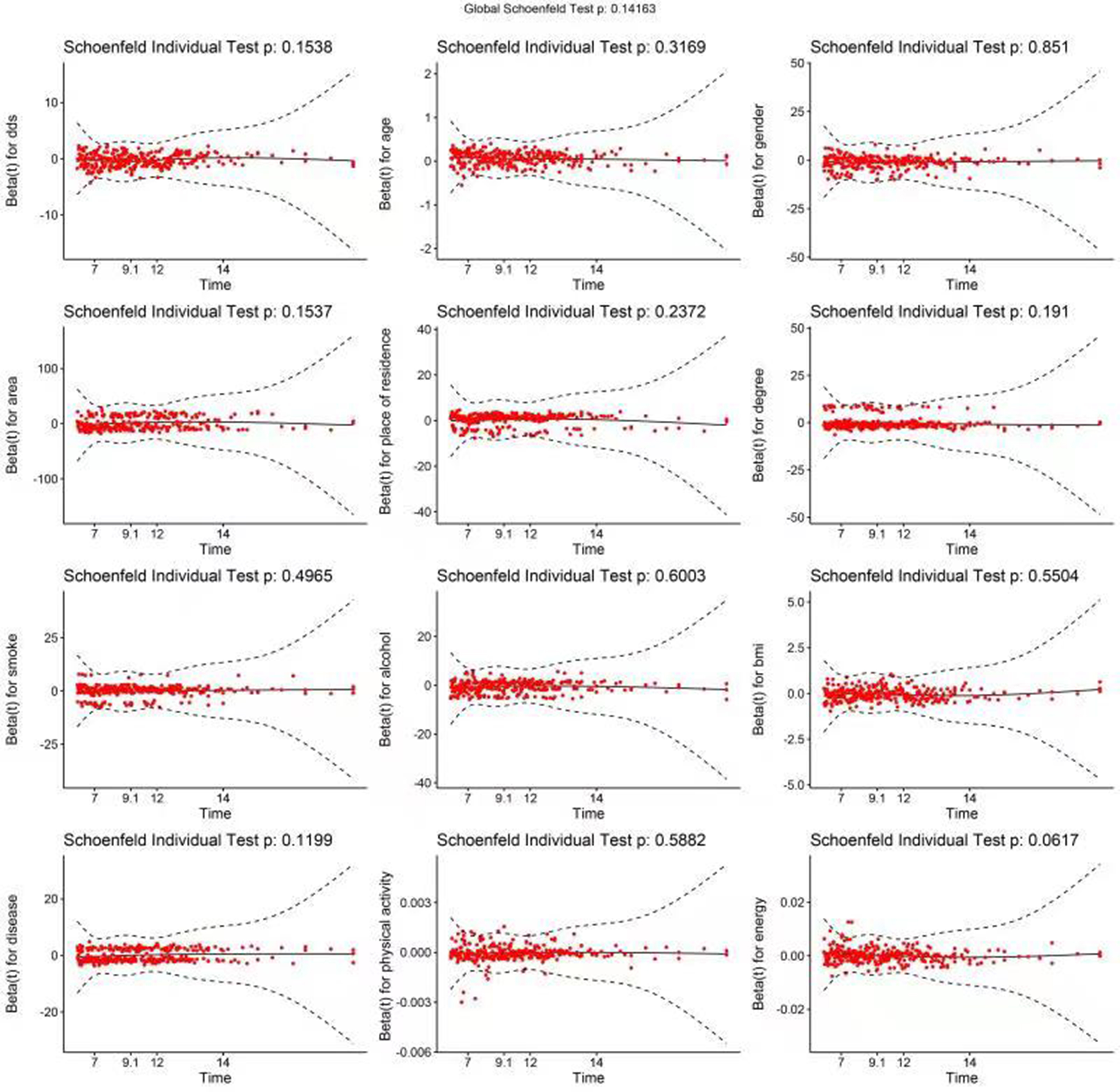

Supplement: Supplementary Figure S2 — Proportional hazard assumption of covariates for the Cox regression of average dietary diversity score and mortality according to the Schoenfeld residuals test, and the results showed that all the covariates met the assumption based on a p-value threshold of 0.05 (P = 0.14). [file Image_2.jpg]
